# Crimson Spotted Rainbowfish (*Melanotaenia duboulayi*) Change Their Spatial Position according to Nutritional Requirement

**DOI:** 10.1371/journal.pone.0148334

**Published:** 2016-02-05

**Authors:** M. J. Hansen, T. M. Schaerf, J. Krause, A. J. W. Ward

**Affiliations:** 1 Animal Behaviour Lab, School of Biological Sciences, the University of Sydney, Sydney, NSW 2006, Australia; 2 School of Science and Technology, University of New England, Armidale, NSW 2351, Australia; 3 Department of Biology and Ecology of Fishes, Liebniz-Institute of Freshwater Ecology and Inland Fisheries, 12587 Berlin, Germany; 4 Humbolt University of Berlin, Faculty of Life Sciences, Thaer Institute, 8–18 Berlin, Germany; Evolutionary Biology Centre (EBC), Uppsala University, SWEDEN

## Abstract

Decision making in moving animal groups has been shown to be disproportionately influenced by individuals at the front of groups. Therefore, an explanation of state-dependent positioning of individuals within animal groups may provide a mechanism for group movement decisions. Nutritional state is dynamic and can differ between members of the same group. It is also known to drive animal movement decisions. Therefore, we assayed 6 groups of 8 rainbowfish foraging in a flow tank. Half of the fish had been starved for 24h and half had been fed 1h prior to experimental start. Groups were assayed again one week later but individuals were allocated to the opposite nutritional treatment. During the assay the positions of individually identified fish were recorded as were the number of food items they each ate and the position within the group they acquired them from. Food-deprived fish were more often found towards the front of the shoal; the mean weighted positional score of food-deprived fish was significantly larger than that of well-fed fish. Individuals were not consistent in their position within a shoal between treatments. There was a significant positive correlation between mean weighted positional score and number of food items acquired which displays an obvious benefit to front positions. These results suggest that positional preferences are based on nutritional state and provide a mechanism for state-dependent influence on group decision-making as well as increasing our understanding of what factors are important for group functioning.

## Introduction

Social animals are known to obtain clear benefits from group membership, however, the costs and benefits obtained by each individual within the group vary according to their relative spatial position in that group [1–3]. The precise positional costs and benefits are determined by the biotic environment, however it is generally recognised that animals at the front of moving groups, and at the periphery of stationary groups achieve higher rates of food intake, at the cost of greater predation risk [4– 8].

A far-reaching and common example of unequal fitness returns due to spatial position is the theory of marginal predation, where the prediction is that if predators attack the closest prey, individuals towards the outside of the group are under higher risk of predation than those towards the centre of the group [7, 9–12]. For moving animal groups the predation threat is higher at the front edge of the group as they are the first to enter new environments and to encounter ambush predators [7, 13, 14]. In many environments, the periphery of animal groups is also where food reward is greatest, either in the quantity or quality of food items or because of reduced competition [4, 10, 15–20]. However, this depends on group size and the particular distribution of food items, as when food items are quickly depleted, individuals at the front of the group will have the highest intake rates. When food items are more slowly depleted, within-group position may be less important, and rather dominance or size may decide which group member has the highest intake [21]. Therefore, positions within the group are often seen as a simultaneous balance between these two forces; predation risk and feeding reward [1, 8, 21–24]. Besides predation risk and feeding reward, a third major factor affecting spatial positioning is energy expenditure, particularly for moving animal groups [25, 26]. Individuals at the leading edge of animal groups may be exposed to greater forces of friction than those behind in the slipstream [27–31] and may even cover greater distances when travelling [32]. Individuals may also position themselves within a group depending on individual laterality [33] or to reduce exposure to adverse environmental conditions such as harsh temperatures [34–36].

An individual’s spatial position may affect the degree to which it influences group movement and decision-making [37–40]. Individuals at the front of moving groups often have greater influence on the direction of movement [13, 41, 42, 43] and it has been shown that a small minority of individuals can direct large groups [37, 44–51]. Many groups are composed of individuals that hold varying amounts and different types of information. In these situations, individual spatial positioning within the group becomes a method in which individuals can exert influence over the group. Physiological demand, personality and parasitism are all aspects that may affect motivation and the likelihood of being at the front, where they are able to exert disproportionate influence on group movement decisions [52–62].

In many stable, restricted entry animal groups, positioning is affected by predation risk, food rewards and energetic requirements, but it is also strongly affected by dominance hierarchies and individual affiliations [6, 63–67]. However, many animal groups are open entry systems, where group membership is temporary and group composition is therefore dynamic. In such groups, dominance relationships are thought to have less of an influence than effects such as size, metabolism and internal nutritional requirements [32]. Within fish shoals, spatial positions are hypothesised to result from differences in size [68], speed [69], parasitism [62], predation threat [7] and internal stimuli such as nutritional state. Hungry fish are often more spread out than satiated fish [70–73] presumably to reduce competition for food, and are often found at the front of shoals [4, 6, 18] (roach, *Rutilus rutilus*), where they have better access to food [74], (*Caranx ignobilis*; [4, 18], roach; [68], *Gadus morhua*). Of the studies performed under controlled conditions, Krause et al. 1992 looked at small shoals of 2 or 4 roach and found that food-deprived fish (2–6 days) were more often to be found towards the front of the shoal than well-fed fish. Similarly, roach deprived of food for 3 days [18] and for 7 days [6] were more often towards the front of the shoal. Whilst these experiments have been highly influential there is need to see if individual fish change positions according to their nutritional demand after shorter, more ecologically relevant, periods of food deprivation and group sizes [75], with the same fish moving toward the front when hungry and toward the back when satiated. Also, there is a need to record how much fish eat whilst in different positions over multiple consecutive foraging opportunities to calculate the direct costs and benefits to different positions. Doing so will provide evidence on whether positional preferences are dynamic and based primarily on nutritional state rather than more consistent individual differences such as size, metabolic rate or behavioural syndrome.

To address this question, we conducted a repeated measures experiment on 6 groups of 8 individually identifiable crimson-spotted rainbowfish (*Melanotaenia duboulayi*). Rainbowfish were chosen as a system to study the impact of nutritional state on spatial dynamics as they live in open entry systems, where interactions are less constrained by a hierarchy. Although there is evidence of dominance hierarchies amongst males [66] we did not see signs of aggressive interactions during the trials and do not believe that dominance was important in the context of this experiment. Rainbowfish were also chosen as they are large enough to monitor intake rates and yet small enough to form shoals in laboratory conditions. They also align with the current and can encounter high flow rates in the wild [67]. They form small shoals of 2–20+ fish and whilst they often swim cohesively as a shoal, they undergo regular fission and fusion events and individuals or smaller groups may break away temporarily and form shoals of a different composition of individuals. Fish were assayed swimming and feeding within a water flow tank to constrain fish shoals to swim in a stable area under the frame of the camera. The same individuals were assayed on two separate occasions, one week apart, in mixed shoals of well-fed and food-deprived fish (1:1). Each fish was assayed twice, once when food-deprived and once when well-fed, but always in the same group composition. This approach avoids confounding effects due to individual differences. Data was collected on their spatial position within the group and also how many food items they consumed and from what positions they attained the food. It was hypothesised that starved individuals will move towards positions that result in them consuming the greatest proportion of food items and this is predicted to be towards the front of the shoal [18].

## Materials and Methods

### Experimental animals

Crimson-spotted rainbowfish, *Melanotaenia duboulayi*, are a freshwater species of fish endemic to eastern Australia. Experimental fish were obtained from Pisces Aquatics and kept in white plastic housing aquaria (180 L) in de-chlorinated aged tap water with a sponge filter at 27°C for 10 weeks in 12:12 light:dark photoperiod before the commencement of experiments. Fish used in the experiment had a body length of 50±5mm. Forty-eight fish were taken from the 180L aquarium and separated into groups of 8 and placed in six separate 50L holding aquaria two weeks before the experimental assay. Fish in each of the six 50L aquaria were anaesthetised with clove oil and tagged with visible implant elastomer (Northwest Marine Technology, Inc, Manual Elastomer Injection System, 10:1 Formulation) on their dorsal surface for individual recognition. During these two weeks all fish were fed live Chironomid larvae once per day till satiation. This study and protocol was approved by the University of Sydney Animal Ethics Committee (Permit Number: 2013 5735).

### Experimental arena

The experimental arena was a rectangular flow tank (3000 × 450 × 100 mm) composed of grey Perspex 5mm thick. De-chlorinated aged tap water at the same temperature as the holding aquaria water entered one end of the flow tank through a plastic hose (30mm diameter) connected to a t-junction of PVC pipe (52mm diameter). This t-junction had 46 holes (10mm diameter) in two rows drilled into one side of it from which the water flowed out. The water then passed through a wall of white Corflute^®^ (100 mm) before entering the experimental arena. Corflute^®^ is a non-toxic Polypropylene sheeting that was used to ensure the water flow was even across the width of the arena. This wall of white Corflute^®^ defined the frontmost barrier to the experimental arena and an identical wall of white Corflute^®^ defined the rearmost barrier. Experimental fish were therefore unable to escape from either end of the experimental arena; the dimensions of the area of water accessible to fish was 1120 × 440 (× 100) mm. White plastic was attached to the base of the arena between these two barriers to allow for better contrast for fish identification.

**Fig 1:**
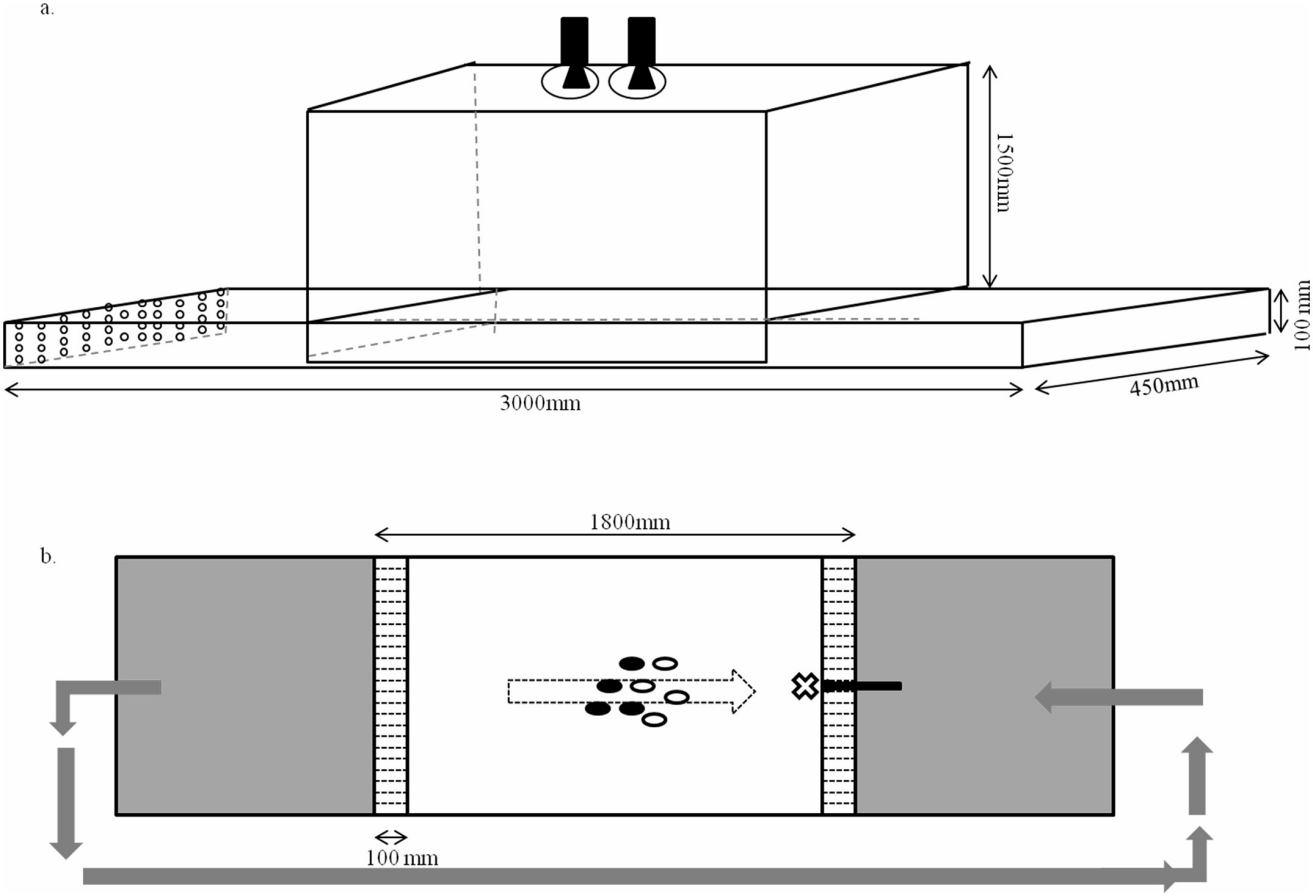
Diagram of the experimental arena, a rectangular flow tank showing a.) its dimensions and the positions of cameras sitting upon the custom made metal frame and b.) the direction of water flow, the location where food items were released from the hose (X) and the dimensions of the area of water accessible to fish defined by the Corflute^®^ barriers. The dotted arrow shows the angle of orientation of the shoal into the current and the fish are identified as food-deprived (white ellipses) and well-fed (black ellipses).

This area was also surrounded by a custom built metal frame that was surrounded by white Corflute^®^ to minimise external stimuli disturbing the fish whilst also allowing enough light for video recording (Fig 1). The water, after passing through the rear most wall of Corflute^®^ fell through 52 holes (10mm diameter) drilled into the end and sides of the flow tank and into a 150L white plastic tub. This water was then pumped back up (Laguna PJ MAX-FLO 18000, 160W) through the hose into the t-junction and continually circulated through the tank at a constant depth of 60mm and constant flow (rate 0.1m/s). Evenness of the current was tested prior to the experiment using green food dye. Food could be injected into the water current by syringing 15ml of water through a small plastic hose that was filled with water, and contained a food item. The hose was on the outside of the front most wall, which meant fish could not see food until it entered the water column and drifted towards them, whereupon the fish would make an attack on the food item. This method also insured that the injection of the food item was not associated with the release of air bubbles or any other stimulus. Fish aligned to the direction of the current, so the direction of the food source corresponded to the front of the group. A video camera (Canon AVCHDProgressive LEGRIA HFG30) and a single lens reflex camera (Canon G1x Powershot) were attached above the experimental arena for data collection.

### Experimental trial

The day prior to data collection a group of 8 fish were placed in the flow tank at 17:00h and allowed to explore and get used to their surroundings overnight. A sheet of white Corflute^®^ was placed on top of the tank to stop fish from jumping out of the arena. At 9:00h the following morning, 5.5 hours before the trial, the cover was removed so fish had time to get used to the change in their environment before the trial began. At 12:00h, two glass aquaria (300x150x150mm) were placed side by side lengthwise in the middle of the flow tank. The adjoining wall of these tanks was a perforated plastic divider that allowed fish to see and smell fish on either side of the barrier, however, it prevented fish physically crossing from either side so that two sub-groups of fish could be fed separately. Four fish were placed in one aquaria and 4 fish in the other. Fish could be manipulated to be in one of two nutritional states: food-deprived or well-fed. Food- deprived fish were not fed, which meant they had been starved for 24h. Well-fed fish were fed with Chironomid larvae at 13:00h via a tube that extended outside of the arena’s white opaque walls till they were satiated. The side that fish were fed was randomized. At 14:00h the glass aquaria were removed, releasing the fish back in to the experimental arena where they promptly formed a shoal facing into the current. After 30 minutes the video was started, and on the half minute mark of each minute a stillshot was taken remotely with the camera over a period of 30 minutes for fish identification. This did not disturb the fish. A Chironomid larva was released from the feeding tube (located in the middle of the front wall (see X on Fig 1b)) into the arena after 5 minutes where upon it drifted downstream toward the shoal. This was repeated 24 times, once a minute, before the film was stopped and the fish removed. The flow tank was then emptied, cleaned, and refilled before another group of fish was placed in the arena at 17:00h for assaying the next day. Each group was assayed twice, one week apart. Each individual was assayed once when well-fed and once when food-deprived but group composition remained consistent between trials. Each group was placed into the flow tank for 24h one week before they were assayed the first time, to familiarize themselves with the experimental set-up.

### Data collection and processing

It was not possible to read the individual fish identification tags from the video alone, however, by matching the video to the 24 still images we were able to determine the rank- order position of each fish in its group, relative to the front end of the flow tank every thirty seconds for 24 minutes. Additionally, we recorded the position of all fish once per minute during the 5 minutes prior to the release of food and one final time one minute after the release of the final food item (in total 54 position ranks were determined for each fish during each trial). We determined the number of times that each fish, *i*, occupied each possible position in the group, *j*, during each trial (with *j* = 1, …, 8; position 1 corresponded to the fish with the greatest *x*-coordinate, at the front of the group–*x*-coordinates increased in the upstream direction). We then determined the proportion of times each fish occupied each possible position via *ρ*_*i*,*j*_ = *n*_*i*,*j*_/54, where *n*_*i*,*j*_ was the number of times that fish *i* occupied position *j*. We assigned each fish a weighted position score for a given trial via $w_{i}\;=\;\sum_{j\;=\;1}^{8}\left(9\;-\;j\right)\rho_{i,\;j}\;$. In addition, we calculated weighted position scores for individual fish for the five minutes that preceded the release of food. This meant that a higher weighted positional score was representative of an individual that occupied frontal positions more frequently. Also from the video, we identified the fish that consumed each food item. The 24 still images were imported to a tracking program, Image J, wherein the (*x*, *y*) coordinates in pixels of each individually identified fish was taken from the point of its snout for each of the 24 images. These coordinates were then imported into MATLAB [76]. In addition, for each trial we recorded the coordinates of the food source in pixels (from a single image), and four reference points from the experimental arena in pixels (the top left, top right, bottom right and bottom left corners of the area accessible to the fish). We used the four reference points and the known dimensions of the area accessible to the fish (1120 mm in the *x*-direction, and 440 mm in the *y*-direction) to obtain four estimates for the number of pixels per millimetre. We used the mean of these pixels per millimetre estimates to then convert the (*x*, *y*) coordinates of each fish and the food source to millimetres for each trial. We denoted the coordinates (in mm) of each fish, *i*, in image *t* for a given trial as (*x*_*i*_ (*t*), *y*_*i*_ (*t*)) and the associated coordinates of the food source as (*f*_*x*_ (*t*), *f*_*y*_ (*t*)). For visualisation purposes, we produced smoothed plots of the relative frequency that hungry and satiated individuals occupied different positions within each group. To do this, we first shifted the coordinates of all fish in all images for all trials to a standard coordinate system where the origin was defined at the group’s centroid for a given frame, and the direction of water flow was parallel to the *x*-axis (moving in the negative *x*-direction). For each image in each trial the group’s centroid, (*c*_*x*_ (*t*), *c*_*y*_ (*t*)), was given by the mean *x*- and *y*-coordinates of all group members. We then shifted the coordinates of all fish for each image according to: *x*_*s*, *i*_ (*t*) = *x*_*i*_ (*t*)-*c*_*x*_ (*t*) and *y*_*s*, *i*_ (*t*) = *y*_*i*_ (*t*)—*c*_*y*_ (*t*). (This shifting placed the group centroid at the origin at time *t*.) The overhead cameras were aligned with the flow tank such that water flowed from the right to the left in each image, parallel to the horizontal axis of each image.

We separated group members and their associated sets of shifted coordinates into sets corresponding to food-deprived fish and well-fed fish. We then processed our data to produce smoothed versions of relative frequency histograms of individual positions relative to group centroids. Smoothing was achieved through the following steps. We divided a portion of the domain centred on group centroids into a set of overlapping square 20 mm × 20 mm bins such that the left edges of the bins were located at *x*_*l*, left_ = -200, -195, -190, …, 200 (mm), the right edges of the bins were located *x*_*l*, right_ = -180, -175, -170, …, 220 (mm), the bottom edges of the bins were located at *y*_*k*, bottom_ = -200, -195, -190, …, 200 (mm) and the top edges of the bins were located at *y*_*k*, top_ = -180, -175, -170, …, 220 (mm). We tallied the number of times food-deprived fish and well-fed fish were located in each bin, indexed (*l*, *k*), across all 12 trials (fish *i* was located in bin (*l*, *k*) in frame *t* if *x*_*l*, left_ < *x*_*s*, *i*_ (*t*) *≤ x*_*l*, right_ and *y*_*k*, bottom_ < *y*_*s*, *i*_ (*t*) *≤ y*_*k*, top_). We stored the tallies for food-deprived and well-fed fish in two separate matrices. The use of overlapping bins to smooth position data is standard in studies of animal behavior (see for example [77]); a consequence of such smoothing is that each fish likely occupied multiple bins in each image, but the binned data was not used in any subsequent inferential tests. We converted the tallies stored in the matrices for food-deprived and well-fed fish into relative frequencies by dividing the value of each element in a given matrix by the sum of all elements in the same matrix. We then rendered the relative frequencies as a function of position relative to group centroid as surface plots using MATLAB’s intrinsic *surf* function. In addition to the surface plots, we constructed smoothed line graphs of the relative frequency that fish occupied different locations examining either *x* or *y* coordinates separately to clarify if fish in different states were positioned differently relative to their group’s centroid, parallel or perpendicular to flow direction. To do this, we allocated data into bin *l* if *x*_*l*, left_ *< x*_*s*, *i*_ (*t*) *≤ x*_*l*, right_, irrespective of the fish’s corresponding *y* coordinate for a smoothed plot of relative frequency as a function of *x*. Similarly we allocated data to bin *k* if *y*_*k*, bottom_ < *y*_*s*, *i*_ (*t*) *≤ y*_*k*, top_, independent of *x*_*s*, *i*_ (*t*) to generate a smoothed plot of relative frequency as a function of *y*. To complement analysis of weighted positional scores, we used the coordinate data imported into MATLAB to construct a bar graph. The bar graph illustrated the proportion of food eaten from different positions, based on distance to food items rather than *x*-coordinate. The bar graph also displayed the fraction of the food taken by food-deprived or well-fed fish. Distances were determined using the standard formula $d_{i}(t)\;=\;\sqrt{{\left(f_{x}(t)\;-\;x_{i}(t)\right)}^{2}\;+\;{\left(f_{y}(t)\;-\;y_{i}(t)\right)}^{2}.}$ Fish closest to the food source were assigned rank 1 for a given time step, *t*, up to the fish farthest from the source that was assigned rank 8. Distance ranks were not weighted or combined for individual fish for the bar graph.

### Statistical analysis

We used randomisation methods (see for example [78]) to estimate probabilities that differences in weighted position score for fish randomly assigned to one of two sets would be greater than the observed difference in weighted position scores for food-deprived versus well-fed fish. The randomisation analysis was applied separately to data corresponding to the entire 30 minutes of observations (across all 12 trials) and data corresponding to the 5 minutes prior to food being made available (across all trials, across trials during the first assay only and across trials during the second assay only). Within each trial we first summed the weighted position scores of all food-deprived fish,$h\;=\;\sum_{\text{hungry}}w_{i}$, and the weighted position scores of all well-fed fish, $s\;=\;\sum_{\text{satiated}}w_{i}$. We then summed the differences *h* − *s* across all *k* = 12 sets of observations to determine a reference test statistic, $F_{\text{ref}}\;=\;\sum_{k\;=\;1}^{12}\left(h_{k}\;-\;s_{k}\right)$. Next, we decoupled the weighted position scores of fish from their manipulated internal state. We randomly assigned four fish in each trial to be nominally food-deprived during a given group’s first assay, with the other four fish assigned a nominally well-fed state. If a fish was assigned a nominally food-deprived state during its first assay, then its state was set as nominally well-fed during the second assay and vice versa. (This method of assigning nominal states was chosen to account for consistency in individual behaviour across both assays.) Once all fish were assigned nominal states, we summed the weighted position scores of all nominally food-deprived fish within a trial, $h^{\prime }\;=\;\sum_{\text{nominally hungry}}w_{i}$, and the weighted position scores of all nominally well-fed fish, $s^{\prime }\;=\;\sum_{\text{nominally satiated}}w_{i}$. We then summed the differences *h'* − *s'* across all trials to form another test statistic, $F_{\text{rand}}\;=\;\sum_{k\;=\;1}^{12}\left({h^{\prime }}_{k}\;-\;{s^{\prime }}_{k}\right)$. We repeated this procedure of randomly assigning fish nominally food-deprived or nominally well-fed states through to calculation of *F*_rand_ one million times. We then calculated $P\left(F_{\text{rand}}\;>\;F_{\text{ref}}\right)\;=\;\frac{\text{number of times }F_{\text{rand}}\;>\;F_{\text{ref}}}{1000000}$; an estimate for the probability that a larger difference in weighted position scores could occur for randomly categorised fish than for fish that had been manipulated to states food-deprived or well-fed. We applied the same randomisation procedure as described above for data corresponding to the first 5 minutes of each trial (before food was available), with the exception that there was no need to account for consistency in individual behaviour when we applied the analysis to data from the first and second assays separately.

We examined if there was a simple relationship between the weighted position scores of each fish during the first and second assays, again using a randomisation procedure. We first determined the absolute difference between the weighted position scores of each fish between each group’s first and second trials. We next determined the mean absolute difference of weighted position scores in each group, and then calculated the mean of the mean absolute difference of weighted position scores across all 6 groups to generate a reference test statistic, *w*_ref_. Randomisation then followed by randomly pairing the weighted position scores of fish within the same group from the first and second trials, calculating the mean absolute difference of weighted position scores in each group and then calculating the mean across all 6 groups of the mean absolute difference of weighted position scores within each group, denoted *w*_rand_. We performed 1000000 iterations of the process of calculating *w*_rand_. We then estimated the probability $P\left(w_{\text{rand}}\;<\;w_{\text{ref}}\right)\;=\;\frac{\text{number of times }w_{\text{rand}}\;<\;w_{\text{ref}}}{1000000}$. A value of *w*_ref_ lower than what might be expected to appear through random association of weighted position scores between fish in their first and second trials would imply that individuals retained some consistency in their positioning between trials.

Finally, we applied correlation analysis within a randomisation framework to examine if there was correlation between weighted position score and the proportion of food items eaten by each fish. We calculated Pearson correlation coefficients, *r*_*k*_, for the weighted position score of individual fish versus the proportion of food items they ate within each trial, *k*, separately. We applied Fisher’s *z*-transformation to the set of correlation coefficients, such that:

$$z_{k}\;=\;\frac{1}{2}\text{ln}\left(\frac{1\;+\;r_{k}}{1\;-\;r_{k}}\right)$$

We then determined the average of the *z*_*k*_ values, denoted $\bar{z}$, and applied the inverse of Fisher’s *z*-transformation to this average to obtain an estimate for the mean Pearson correlation coefficient across all groups:

$$\bar{r}\;=\;\frac{e^{2\bar{z}}\;-\;1}{e^{2\bar{z}}\;+\;1}$$

We used the method outlined in [79] to determine if the mean correlation was significantly different from zero. The test statistic for this method is:

$$Z\;=\;\frac{\bar{z}}{\sqrt{\frac{1}{\sum_{i}\left(n_{i}\;-\;3\right)}}}$$

where *n*_*i*_ is number of data points for group *i*. Significance is then assessed by determining *P*(*X* > *Z*) where *X* is a standard normal distribution (with mean 0 and standard deviation 1). Randomisation then followed in a process analogous to the other tests described above, this time to estimate the probability that the correlation between weighted position score and a permutation of the proportion of food items eaten by each fish within a trial could exceed the observed correlation between weighted position score and number of food items eaten by each fish (using weighted position scores based on all 54 observations of each fish). The reference test statistic for this new randomisation test was the mean correlation coefficient obtained using the transformation based method above. Within each group we then randomly permuted the observed proportion of food items eaten by each fish, determined a correlation coefficient for that groups permuted data, and then obtained an estimate of the mean correlation coefficient across all groups’ permuted data sets (again using the transformation method), denoted ${\bar{r}}_{\text{rand}}$. We performed 1000000 iterations of the randomisation procedure, and then calculated $P\left({\bar{r}}_{\text{rand}}\;>\;\bar{r}\right)\;=\;\frac{\text{number of times }{\bar{r}}_{\text{rand}}\;>\;\bar{r}}{1000000}$.

All statistical analysis was performed using custom codes in MATLAB [76].

## Results

**Fig 2:**
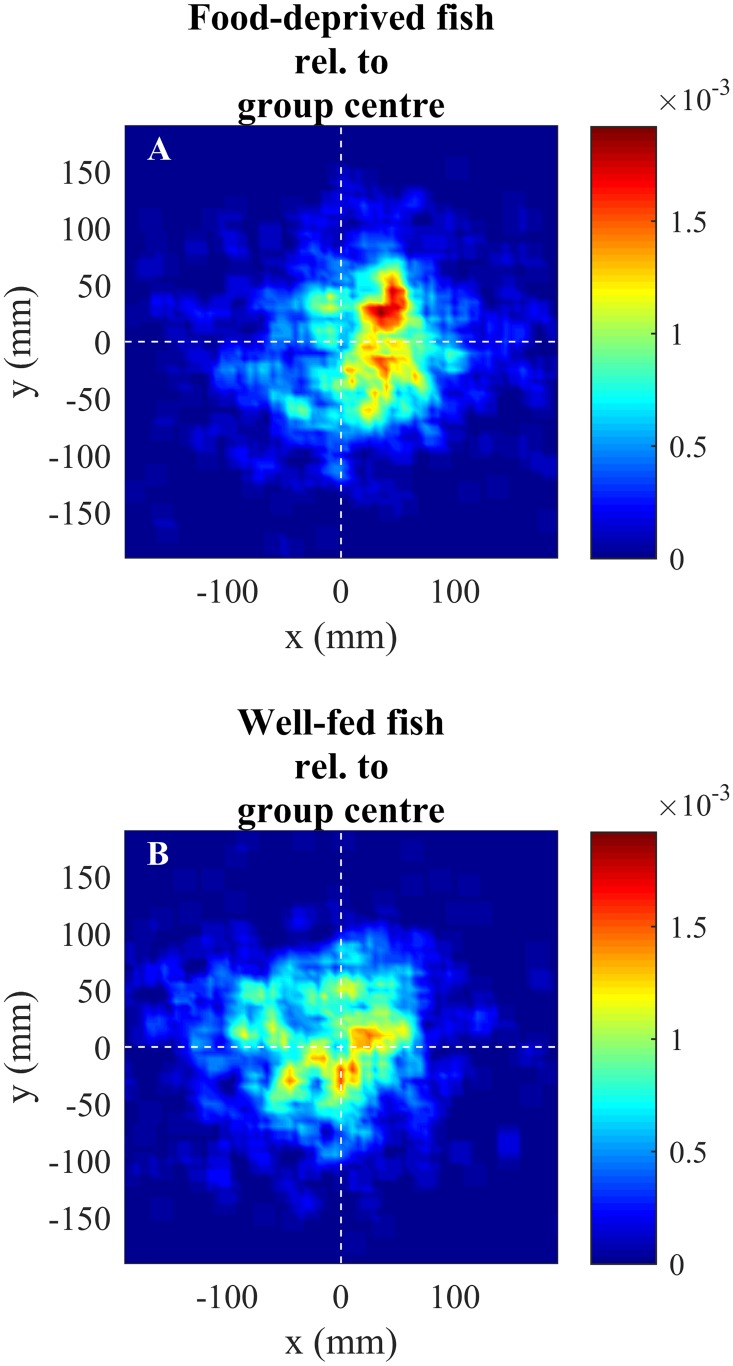
The relative frequency that food-deprived (A) and well-fed (B) fish occupied different spatial locations relative to the group centroid (at (0, 0)). Both plots were smoothed through the use of overlapping square bins (see *Data collection and processing* for more details).

**Fig 3:**
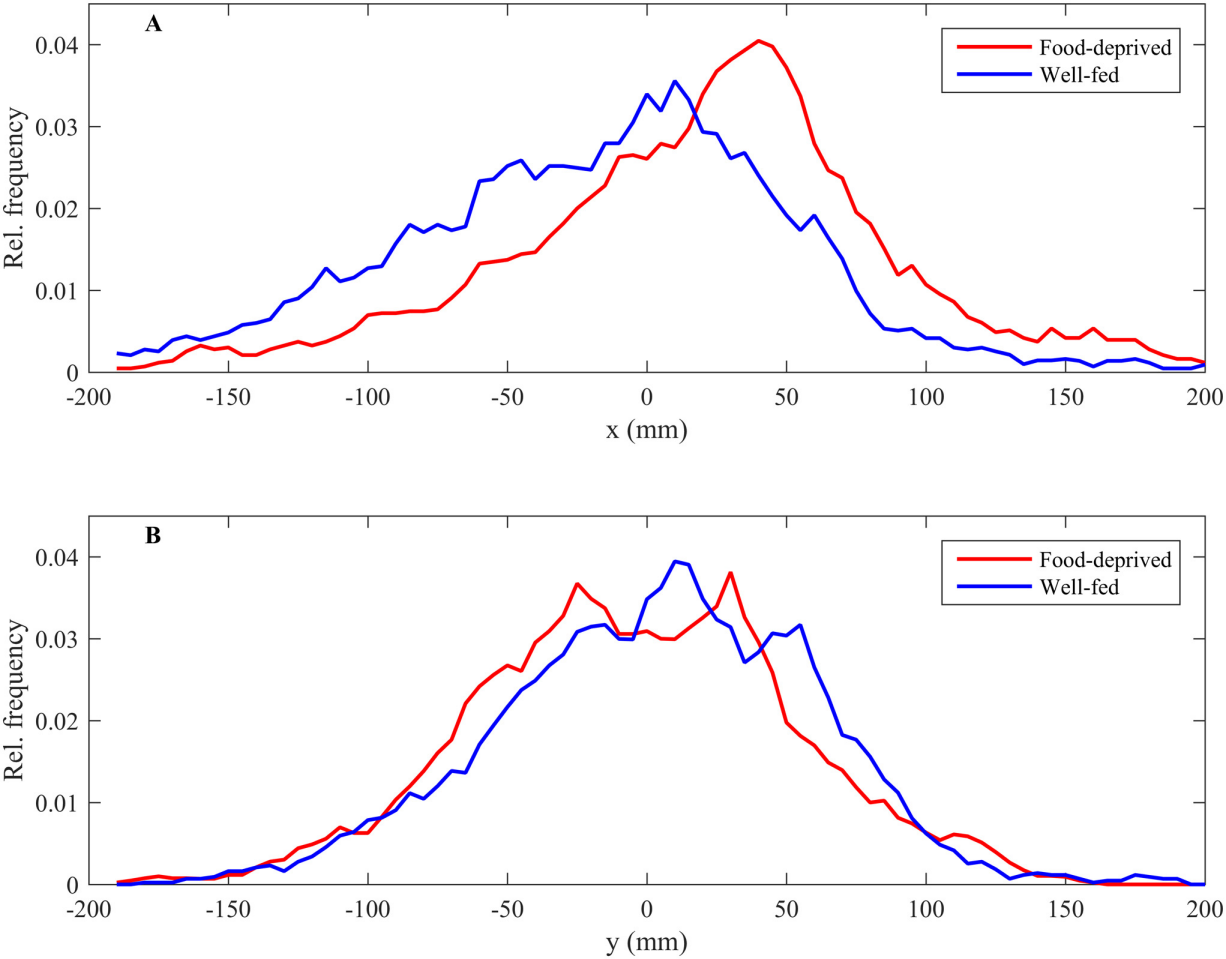
The relative frequency that food-deprived (red lines) and well-fed (blue lines) fish occupied different *x*- (A) or *y*-coordinates (B) relative to the group centroid (at 0).

The weighted positional score of food-deprived fish was significantly larger than that of well-fed fish (*F*_ref_ = 66.482, p ≈ 0 from randomization analysis with 1000000 iterations) with food-deprived fish more frequently occupying positions at the front of the shoal compared to well-fed fish (Figs 2 and 3).

The effect of nutritional state on the weighted positional score over the first 5 minutes of the experiment, before food was made available to the shoal, similarly showed that food-deprived fish occupied positions at the front of the shoal more frequently than well-fed fish (*F*_ref_ = 46.600, *p* < 0.001 from randomization analysis with 1000000 iterations). However, when data corresponding to the period before food was made available for the two trials was analysed separately, we found that there was no effect of nutritional state on mean weighted positional score in the first trial (*F*_ref_ = 15.000, *p =* 0.09, 1000000 iterations). In the second trial, after fish had experienced feeding in the flow tank food- deprived fish occupied positions at the front of the shoal more frequently than well-fed fish (*F*_ref_ = 31.600, *p =* 0.001, 1000000 iterations). The observed mean absolute difference in weighted position scores for fish between their first and second assays was sufficiently large that it did not differ statistically from random (*w*_ref_ = 1.530 *p =* 0.207, 1000000 iterations) which suggests individuals were not consistent in their position within the shoal between treatments.

**Fig 4:**
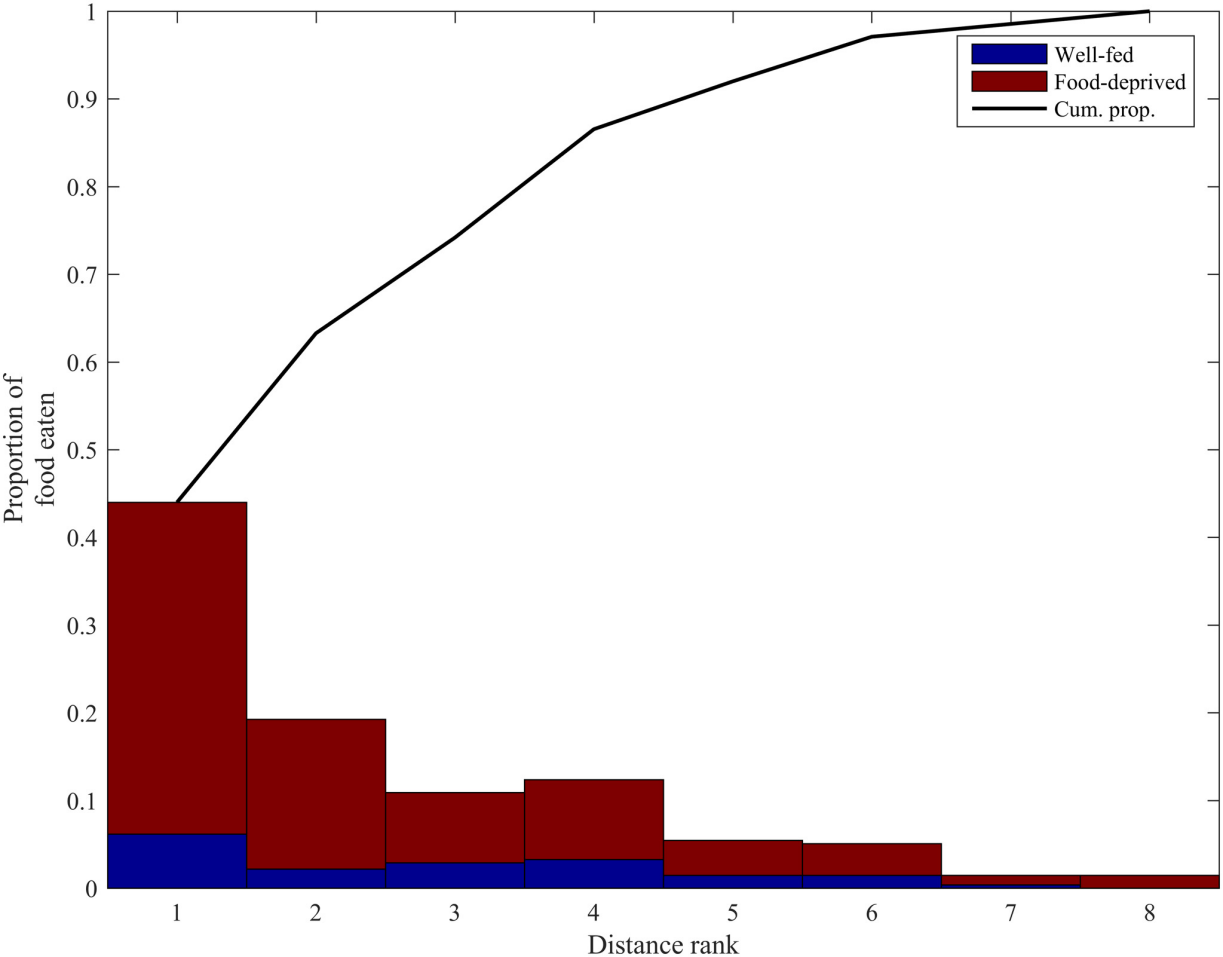
The proportion of available food eaten by fish with different distance ranks (determined by distance to food source: 1 = closest to food, 8 = farthest from food), divided into food-deprived (red) and well-fed (blue) individuals. The solid black line indicates the cumulative proportion of food eaten as a function of increasing distance rank.

There was a significant positive correlation between mean weighted positional score and proportion of food items eaten ($\bar{r}\;=\;0.748$, Z = 7.507, *p <* 0.001). Subsequent randomization analysis suggested that the observed correlation was unlikely to occur through random association of the proportion of food items eaten with weighted position scores of fish within each trial (p ≈ 0 from randomization analysis with 1000000 iterations). Additionally, more food items were eaten by fish closest to the food source than those located at greater distances; fish with distance ranks 1 and 2 consumed a cumulative proportion of available food items of over 60% (Fig 4).

## Discussion

As predicted, food-deprived fish occupied the front most positions of the shoal more frequently than well-fed fish. This is evidence that positional preferences were based primarily on nutritional state and less so by individual differences such as size, metabolic rate or behavioural syndrome, as the same individuals were tested twice in the same group composition, once when well-fed and once when food-deprived. Also, individuals were not consistent in their position within the shoal between treatments. This finding supports existing literature that proposes individuals position themselves within groups according to nutritional state [4, 6, 18, 80]. For example, hungry whirligig beetles, (Coleoptera: Gyrinidae) position themselves on the periphery of the group and spatially separate themselves from near-neighbours in order to obtain the majority of food items [8].

In fish shoals, it has been suggested that food deprived fish more often occupy the frontmost positions within a shoal due to a higher swimming speed or turning rate, and indeed fish do slow down as they become satiated [81]. However, as the shoals were tested within a flow tank they were restricted from swimming much faster or slower than conspecifics (due to the barriers at the front and back of the tank). The results of this experiment suggest that spatial positions may result from individuals positioning themselves in relation to shoal mates, highlighting the importance of conspecifics on the spatial behaviour of individuals within groups, rather than solely individual traits such as speed. This positioning effect was perhaps heightened by competition for food items that entered the arena from directly in front of the shoal’s facing direction. However, although the effect size was smaller, the effect of nutritional state on the weighted positional score over the first 5 minutes of the experiment, showed that food-deprived fish more frequently occupied the front of the shoal. This section of the assay occurred before food was made available to the shoal, but after they had experience feeding from food drifting towards them in the first trial. In the first 5 minutes of trial 1, when fish had no experience of food in the environment, food-deprived fish were not more frequently found at the front of the shoal. These results may be interpreted as food-deprived fish acting on learned information on food location, or that fish in the first trial were less familiar with the environment and therefore were attempting to reduce predation risk or save energy. However, the latter is unlikely as the fish were acclimated to the tank for a long time and ate the food that was made available to them in trial 1, suggesting they were comfortable. It could be argued that fish only occupy frontal positions when they know this is where food will be located, however, we believe that the results show a general trend for food-deprived fish to occupy front positions as they re-enforce earlier findings, which showed, in static water and with no food available, food-deprived rainbowfish (*Melanotaenia duboulayi*) (starved 48hr) occupied frontal positions in shoals [80]. Therefore, it is possible that the finding that food deprived fish not being found to more frequently occupy the front positions of shoals in the first 5 minutes of the first trial is a false negative as the effect is in the same direction as in second trial, but does not reach the level for significance.

Similar effects of food deprivation were found to occur in shoals of 2 roach starved for 2 or 4 days and in shoals of 4 fish starved for 4 and 6 days [4]. However, Krause et al. 1992 followed a single focal fish within small shoals, whilst this experiment was able to account for the position of all 8 shoal members at each time interval. This is an important advancement as decisions made by an individual pertaining to foraging behaviour are greatly influenced by the actions of other group members [53]. Also, the time of food deprivation in Krause et al. 1992 was, at its minimum, twice as long as in the current experiment. Further experiments on roach, also had longer food deprivation periods of 3 [18] and 7 [6] days. To our knowledge, 24h is the shortest food deprivation period known to have a significant effect on individual spatial positioning in fish shoals and provides strong evidence that spatial positioning based on internal nutritional state is more sensitive than previously known. This is important as short-term changes in hunger levels are more likely as these animals feed frequently; therefore larger differences between individuals are not as likely to occur.

The frequency that food-deprived fish occupy frontal positions is known to increase with food deprivation [4] and fish in the wild lose the preference for frontal positions after 2 days of being allowed to forage freely [18]. Unfortunately, the difference in the hunger levels between fish that started the trial food-deprived and fish that started the trial well-fed did not alter sufficiently to see a rotation of positions during the 30 minute trial. This was likely a combination of not introducing sufficient food for the food-deprived fish at the front to become satiated and not having a long enough trial duration for the well-fed fish at the back to become hungry. The mechanics behind the rotation of spatial positions based on dynamic changes in internal state is the next logical step for this field of research. It is possible that in this species a rotation of positions would not occur as in more natural systems shoals undergo regular fission and fusion events, breaking up and reforming shoals with new individuals, however, more obligate shoalers and other animals that have more permanent group composition may undergo rotational movements based on internal state. Attempts to explore this may like to use a similar flow tank set-up as it has proved successful in ensuring synchrony of shoal travel direction for long periods of time and allowed for accurate measurement of which individual fish ate and from what position it consumed the food item. However, future experiments should provide more food over longer trial durations and utilize automated tracking software to acquire data at a finer scale than in the current study.

Fish in the two frontmost positions acquired over 60% of the food evidence of a clear benefit to fish that position themselves at the front of the shoal. The majority of available food being consumed by the frontmost individuals is similar to previous experiments calculating the number of food items eaten by individual fish within a similar sized shoal of roach (n = 10, [6]). However, Krause et al. 1998 recorded the position of the fish in the shoal that ate the first and then the second of two Chironomid larvae that were placed into an arena. All fish in the shoal were hungry and the shoal only experienced a single foraging event. In contrast the shoal in the current study was composed of both hungry and satiated individuals and 24 food items were drifted towards the shoal (one at a time) which meant that fish had multiple foraging opportunities and could change positions according to previous success. Therefore, the result is novel in that it records, for the first time, that over multiple consecutive foraging opportunities individual positions within fish shoals are associated with different intake rates and that hungrier fish move to the front where they receive more food.

Other species have similarly showed a difference in intake rate according to spatial position, for example, individual whirligig beetles on the outer periphery consumed almost all of the food made available [8]. A proportion of food items as large as this, however, is likely due to the limited amount of food presented to the shoal at any one time and the temporal dispersion of its introduction to the arena (one item, once per minute in this experiment). Higher food densities at more frequent time intervals would likely lead to fish further towards the rear of the shoal attaining a higher proportion of the available food [21, 82]. In these environments fish at the rear could potentially acquire sufficient food to negate any benefit of moving towards the front or periphery of the group and individual spatial positioning according to nutritional state may not occur under such conditions.

The foraging behaviour of individual fish in shoals has already been shown to be flexible in response to changes in the distribution of food in the environment [83, 84]. Experiments quantifying the amount of food individuals in different shoal positions acquire under different spatial and temporal distributions of food, and how this affects the overall geometry and social dynamics of the shoal is a potentially fruitful area of future research. Individuals in the frontmost positions of moving shoals have a greater risk of predation [7, 13, 14] and may attain further costs in increased hydrodynamic demand [31] (rainbow fish can maintain station in currents over 0.5m/s [67], therefore hydrodynamic benefits of shoaling in this particular species may be minimal at low flow rates.) It is unknown to what extent fish in this study responded to these conflicting demands. A manipulative experiment involving the addition of predator cues to the water in addition to food items as well as a calculation of individual tail-beat frequency could be a useful means of exploring the effects of conflicting demands on spatial positional choice.

The results of this paper are discussed primarily in the context of fish shoals as this is where the vast majority of empirical evidence for spatial positioning based on nutritional state currently exists. However, within-group spatial positioning is known to correspond to different fitness returns in a range of taxa [10, 16, 24, 69], and it is possible that in these groups spatial positioning may also be influenced by nutritional state. In many animal groups leadership or control of group travel direction is determined by select individuals often at the front of the group [13, 37–39] and these individuals may be those that have the greatest motivation, perhaps to seek shelter or attain nutritional balance [52–55, 57–59]. This experiment provides empirical evidence that positions are associated with different intake rates and that differences in nutritional state affect the spatial positioning of individuals within groups, suggesting a mechanism by which individual’s state may influence group decision-making.
